# Study on the additional financial burden of breast cancer disease on cancer patients and their families. Financial toxicity in cancer

**DOI:** 10.3389/fpubh.2024.1324334

**Published:** 2024-07-03

**Authors:** Eduardo J. Fernandez-Rodriguez, Rocio Taboada-Taboada, Alberto Garcia-Martin, Celia Sanchez-Gomez, Susana Saez-Gutierrez, Maria I. Rihuete-Galve, Emilio Fonseca-Sánchez

**Affiliations:** ^1^Deparment of Nursing and Physiotherapy, University of Salamanca, Salamanca, Spain; ^2^Institute of Biomedical Research (IBSAL), Salamanca, Spain; ^3^Deparment of Labour Law and Social Work, University of Salamanca, Salamanca, Spain; ^4^Deparment of Evolutionary and Educational Psychology, University of Salamanca, Salamanca, Spain; ^5^Deparment of Medicine, University of Salamanca, Salamanca, Spain; ^6^Medical Oncology Unit, University Hospital of Salamanca, Salamanca, Spain

**Keywords:** financial toxicity cancer care, healthcare disparities, cost, socio-economic impact, breast cancer

## Abstract

**Introduction:**

Breast cancer is among the most frequently diagnosed cancers worldwide, with 2.3 million new cases reported annually. The condition causes a social and economic impact known as financial toxicity of cancer. The study aims to explore the extra expenses borne by patients and their families on being diagnosed with breast cancer.

**Methodology:**

An observational, descriptive, cross-sectional study was conducted. The data was collected between November 2021 and March 2022 at the Medical Oncology Service in Complejo Asistencial Universitario de Salamanca, Spain. The variables under investigation were additional economic costs, physical disability (as measured by the Barthel Index), instrumental activities of daily living (as measured by the Lawton-Brody Scale), and caregiver burden (measured using the ZARIT scale).

**Results:**

The final sample size was *N* = 107. The study yielded the following outcomes: the median age was 55 years old and the majority of participants were female, with a proportion of 99.1%. The incidence rates for stage I and II were 31.8 and 35.5%, respectively. The median Barthel score was 100 points, while the Lawton and Brody score were 8 points and the ECOG score was 2 points. The analysis of primary caregiver burden resulted in a median ZARIT score of 15 points. The expenses related solely to the cancer diagnosis totaled 1511.22 euros per year (316.82 euros for pharmaceuticals; 487.85 euros for orthopedic equipment; 140.19 euros for home help; and 566.36 euros for housing adaptation or transfer to a hospital). The average annual income before diagnosis was 19962.62 euros. However, after being diagnosed with breast cancer, there is a significant income decrease of 15.91%, resulting in a reduced average annual income of 16785.98 euros. Additionally, a significant correlation was found between total expenditure and the level of dependency (*p* = 0.032) and functional status (*p* = 0.045).

**Conclusion:**

These findings indicate that breast cancer patients experience a considerable economic burden, which worsens as their functional status deteriorates. Therefore, we believe policies should be implemented to help control this economic deterioration resulting from a serious health condition.

## 1 Introduction

Cancer is a widespread ailment with a significant impact on global health. While survival rates have substantially improved over time, cancer diagnosis continues to be one of the primary causes of illness and death worldwide (1).

Cancer is a concern for public health since it influences social, political, economic and cultural transformation. Moreover, breast cancer has a significant impact on the living conditions of a vast segment of society, negatively affecting the quality of life and economic potential of patients and their families due to the substantial social and economic costs associated with the disease (2).

Breast cancer is among the most frequently identified cancers and is expected to affect one in two women during their lifetime. Notably, there were 2.3 million new breast cancer cases globally in 2020 (3), and a total of 34,088 new diagnoses of breast cancer were reported in Spain in 2022 (4).

The cycle of illness affects not only the physical body, but also the personal, family, and environmental spheres. Therefore, a biopsychosocial approach is required. Additionally, the impact of the disease extends beyond the individual and involves their social, familial, and professional surroundings (5).

The patient’s life and family members’ lives will undoubtedly experience alterations throughout the disease’s progression. These changes can impact family and social ties, household duties, and even the patient’s and their relatives’ employment (6).

The adverse effects resulting from cancer treatment, such as nausea, vomiting, dizziness, diarrhea and constipation, can lead to a decline in life quality. In addition to this, the emotional turmoil faced by many patients, encompassing feelings of sadness, anxiety, fear and depression, as well as their social circumstances exacerbate the situation (7).

Cancer patients have diverse physical and psychological requirements throughout their illness and thus necessitate extensive care, including continuous care (8).

The disease’s impact extends to the patient’s ability to reintegrate into the labor market due to periodic absenteeism for check-ups (9).

Cancer remains the most significant socio-health issue, despite its high economic burden on both the patient and their family (10).

The estimated cost of cancer in Spain is €19.3 billion, with breast cancer accounting for €2.2 billion (11). In Spain, the Social Security system covers the expenses of patients’ care. Nevertheless, patients are still responsible for various expenses, including the cost of dietary products, wigs, transportation from home to hospital, alterations to the home, and changes to their diet.

Additionally, it should be noted that this is not the case in all parts of the world, as the costs of the disease largely depend on current healthcare policies. If oncological treatments are not covered by national health systems, the estimated average cost of breast cancer treatment can vary significantly depending on the country, the type of treatment, and the resources used. In the United States, for example, according to a study by the American Cancer Society, the average cost of breast cancer treatment can range from $20,000 to $100,000 per year, depending on factors such as the stage of cancer, the type of treatment, and whether costs of surgery, radiotherapy, chemotherapy, medications, and continuous care are included. In the United Kingdom, the NHS covers most of the treatment costs for residents, but the costs associated with breast cancer treatment (in terms of market value) can be similar to those in other European countries, ranging from £20,000 to £40,000 per year. Meanwhile, in countries with mixed or private healthcare systems, such as some in Latin America or Asia, costs can vary widely. In Brazil, for example, the cost can range from $15,000 to $25,000 per year depending on the type of treatment and insurance coverage.

Cancer has an economic impact on both patients and their family members, as there is a reduction in income that varies based on the length and severity of the illness. This can result in different types of incapacity. In addition, there are costs associated with treatment such as dietary changes and transportation to the hospital.

Illness costs are accepted by families who try to cope as best they can. However, such costs can harm family dynamics, particularly among vulnerable and low-income groups, where expenses increase and income decreases (2). Families bear 45% of the overall costs of the disease, whereas the remaining 55% of these costs are covered by the health care system (11).

Due to time off work because of treatment side effects or the disease itself, patients may experience a severe decrease in income of up to 75%. The disease may have also caused a decrease in income due to disability. Furthermore, there has been a 15% rise in expenses (11).

Breast cancer results in higher household spending on pharmaceuticals, parapharmaceuticals (particularly skin care products), and orthopedic equipment such as wigs, bra fittings, and breast prostheses. Additionally, third-party assistance is required for any task that the diagnosed individual needs help, support, or supervision with. This incurs additional costs (10).

Moreover, there is an income loss for both the affected individual and their caregiver. Breast cancer has a more significant effect on the patient’s income than on the long-lasting costs of the disease, making it challenging to make the financial toxicity of the illness apparent (10).

The economic consequences of cancer result in 24,942 instances of social vulnerability each year solely due to the diagnosis of the disease (11).

Cancer generates a range of needs in patients and families, including social, economic, and employment issues, that are often not considered despite their crucial importance throughout the course of the disease.

We hypothesize that breast cancer patients and their families bear the costs of the disease, which could potentially impact household finances, rather than the Spanish state.

## 2 Materials and methods

### 2.1 Aim and design of the study

An observational, descriptive, cross-sectional, non-probabilistic sampling study was designed without replacement with prevalence of breast cancer disease at baseline.

The aim of the study was to examine the current socioeconomic situation of breast cancer patients at the Salamanca Hospital and to assess the repercussions that may exist depending on the personal situation of each patient.

The study sought to demonstrate that breast cancer patients have difficulties related to the disease in biological terms, but also in economic terms and extraordinary expenses (pharmacy, parapharmacy, support products, help from a third person, etc.).

### 2.2 Participants

Breast cancer patients at the University Hospital of Salamanca were selected for the study according to predetermined inclusion and exclusion criteria.

Inclusion criteria include an oncological breast cancer diagnosis, being a patient at the University Hospital of Salamanca, being over 18 years old, and voluntarily agreeing to participate (by signing an informed consent form).

Exclusion criteria consisted of patients diagnosed with cancers other than breast cancer, those not meeting the age criteria, those who did not consent to participate in the study by not signing the informed consent form, and those who had been previously assessed.

To determine the sample size, we considered the incidence of the disease under study in Spain, in this case breast cancer. In this case, we applied the formula used to estimate the average sample size required. Therefore, we considered that the incidence of breast cancer in the year prior to the study was 34,750 cases, according to the Spanish Society of Medical Oncology (SEOM) (1). Based on this, and assuming a 95% confidence level, with a precision (d) of 3 and a variance (S2) of 250, we obtained a necessary sample size of *N* = 107 individuals. This is adjusted for expected attrition by setting an expected attrition rate (R) of 15%, giving a loss-adjusted sample of 127 individuals.

**Figure 1:**
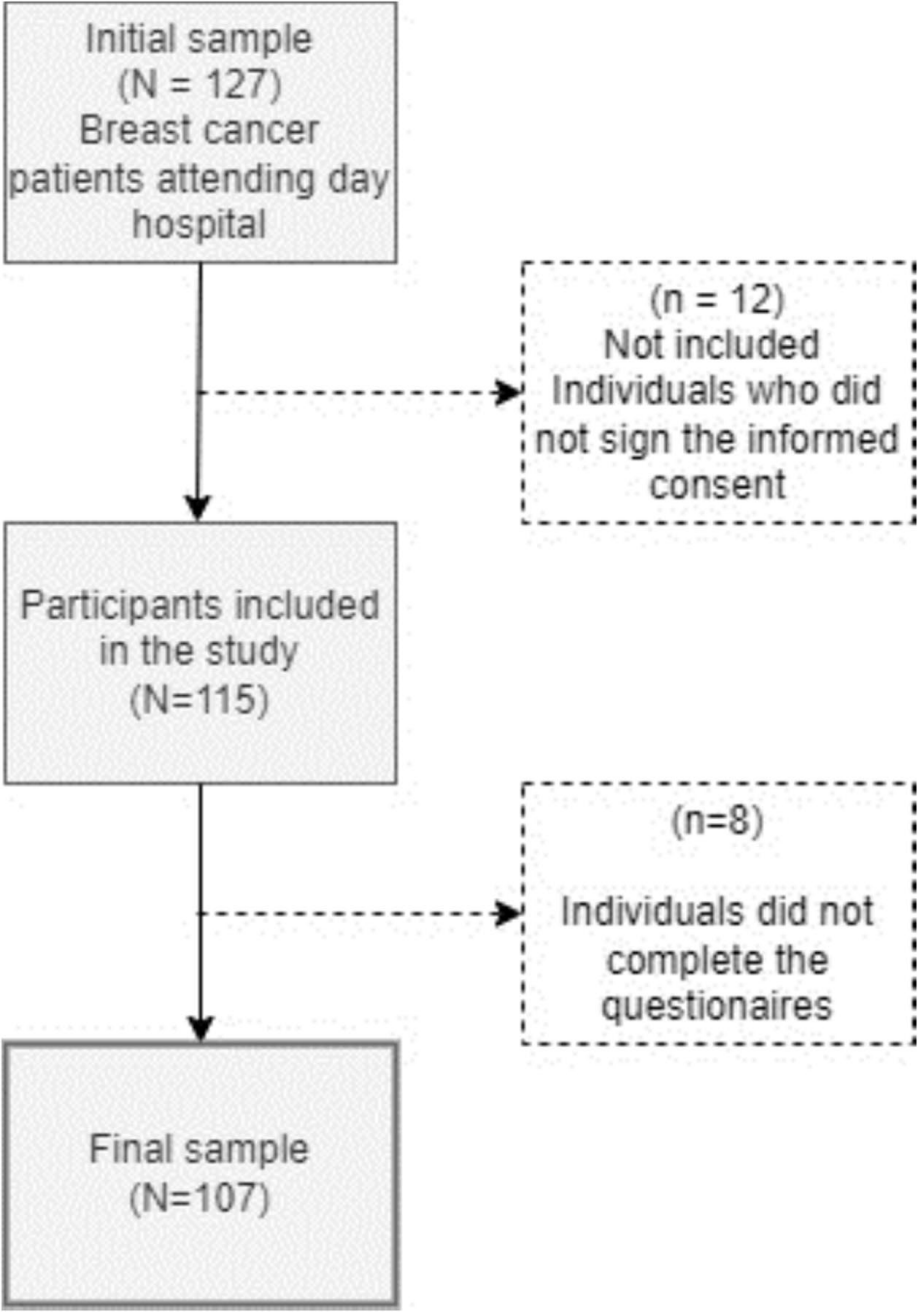
Sample selection flowchart.

A sample of 107 participants was obtained between November 2021 and March 2022. The participants were randomly selected without replacement from patients affiliated with the Salamanca Hospital Complex, including those who were admitted to the Medical Oncology department or receiving outpatient care at the Oncology Day Hospital. Details on the sampling procedure can be found in Figure 1.

### 2.3 Procedure and data collection

The technique used for sample selection was non-probabilistic sampling, simple, without replacement. We used a questionnaire created specifically for this study, which was then passed on to the participants after they had completed the informed consent form. The time taken to complete the questionnaire individually ranged from 10 to 15 min depending on the situation of the patient being interviewed.

After the questionnaire, the patients and caregivers themselves were assessed on different measurement scales chosen on the basis of their reliability and validity to take into consideration the level of dependency, primary caregiver overload and quality of life.

### 2.4 Primary and secondary outcomes

The primary variable under consideration was the supplementary expenses incurred by breast cancer patients and their families, which are not covered by the publicly-funded Social Security or Public Health System. A study-specific questionnaire was employed to quantify these expenses. The secondary variables comprise patient health data encompassing cancer type, date of diagnosis, disease stage, treatment methods, side effects, and level of dependency. We also captured various socio-demographic data and intervening variables related to the employment and financial circumstances of the patient and their family, such as economic standing, employment status, disabilities, pensions/benefits, economic earnings in the last fiscal year, earnings before diagnosis, and changes in family income. We have considered the study of these outcome variables based on two fundamental factors: firstly, the literature review, understanding and extracting from it those factors that could influence our study; and secondly, based on our daily clinical practice, what patients express to us, and our considerations derived from the experience gained in recent years in the Medical Oncology Service.

### 2.5 Variables and measurement instruments

Barthel Index (BI) (12): This tool is used to evaluate patients’ physical disability and assess their functional disability regarding their activities of daily living (ADLs). The BI is highly reliable and valid, and it is straightforward to use and interpret. The scale is divided into 10 items that measure basic ADLs, including eating, washing, dressing, grooming, bowel movements, urination, using the toilet, transferring, ambulation, and walking up and down stairs. Scoring ranges between 0 and 100. The total of scores determines if a patient is classified as Total, having less than 20 score points, Severe, with between 20 and 35 score points, Moderate, with between 40 and 55 score points, Mild, with greater than or equal to 60 score points or fully independent, having 100 score points.

Lawton-Brody Scale (13): This tool assesses independence and dependence in performing instrumental activities of daily living (IADLs). The scale comprises 8 items, including the ability to use a mobile phone, go shopping, take care of the house, do laundry, use means of transport, be responsible for taking medication or drugs, and handle money. Scores range from 0 to 8. The calculation’s outcome can determine the patient’s level of dependency, which may fall under Total (0–1 points), Severe (2–3 points), Moderate (4–5 points), Slight (6–7 points), or Independence (8 points).

ZARIT Caregiver Burden Interview (14): is employed to evaluate stress experienced by the primary caregiver of the patient by means of a 22-item questionnaire with 5 possible responses (never, rarely, quite often, almost always). The responses range from 1 (never) to 5 (always). The total scores may lead to no overload (score of 46 or lower), mild overload (score between 47 and 55), or severe overload (score exceeding 55).

ECOG scale (15): The ECOG scale, also known as the “Performance Status,” assesses the patient’s overall health status and quality of life. It considers the changes in the patient’s daily life activities and is divided into five levels or groups from ECOG 0 (full independence) to ECOG 5 (deceased patient) with only one of the items being scored. Technical terms are explained on first use. Results obtained from this assessment can be: ECOG 0—Patients displaying no symptoms and being able to perform daily activities and exertion normally. ECOG 1—Patients experiencing symptoms that obstruct their exertion but are still capable of carrying out daily activities and light work. The patient is confined to bed only during sleeping hours.

ECOG 2—Patients unable to execute any work due to symptoms and are forced to be in bed for several hours a day along with nighttime sleeping hours, but not more than 50% of the time. The patient is able to meet most personal needs independently. According to the Eastern Cooperative Oncology Group (ECOG) criteria, the patient falls under the ECOG 3 category, requiring to be confined to bed for more than half of the day due to symptoms and requiring assistance with most daily activities. In the ECOG 4 category, the patient remains bedridden and needs assistance with all activities of daily living, including personal hygiene, mobilization in bed, and even feeding. Lastly, in the ECOG 5 category, the patient is deceased.

All measurements were recorded on a data collection sheet for each patient and subsequently entered into a database created specifically for this research.

The instruments necessary to obtain the data were administered on a single occasion and were not carried out sequentially in time.

The study’s objective and the voluntary nature of participation were communicated to the participants and primary caregivers, who authorized their involvement by signing an informed consent form.

The lead researcher provided the patient with the study questionnaire, which was later retrieved and collected by the same individual. The designated measurement scales were then used to obtain the study results.

The questionnaire and measurement scales, based on the sample size and inclusion/exclusion criteria, provided the requisite data to conduct this study.

### 2.6 Statistical analysis

Statistical analysis was performed using International Business Machines’ (IBM) Statistical Package for the Social Sciences (SPSS) version 25 (IBM Corp., Armonk, NY, United States).

We have carried out a descriptive analysis considering maximum and minimum values, as well as the presence of possible outliers, considering or not their suitability by means of a box diagram as a standardized method.

We performed an analysis of the socio-demographic characteristics of the sample and the scores of the instruments and measurement scales of the study.

The variables were analyzed using Kolmogorov–Smirnov statistics by means of which we were able to determine normality by parametric means (normal variables) or non-parametric means (non-normal or ordinal variables).

In all cases we have described the variables with the corresponding statistics. Normally distributed variables have been defined by means of mean and standard deviation using parametric methods. Variables with non-normal distribution have been defined by median and interquartile range following a non-parametric approach.

Categorical or qualitative variables were defined using frequencies and percentages.

#### 2.6.1 Statistical analysis

In all cases we have described the variables with the corresponding statistics. Normally distributed variables have been defined by means of mean and standard deviation (m and s = following parametric methods). Variables with non-normal distribution were defined by median and interquartile range (M and IQR) following a non-parametric approach.

The normality test oriented most of the calculations toward the non-parametric way (*p* < 0.05).

The analysis of correlations was solved with Spearman’s correlation coefficient (Spearman’s rho).

In all cases a 95% confidence interval was considered, i.e., an alpha risk, type I error, set at 0.05 (α = 0.05); with significance indices of *p* < 0.05. The results obtained have been expressed with the value of the statistic, as well as the *p*-values and those data that are most interesting for the interpretation of the results.

Data were analyzed with the SPSS Statistics version 26.0 software (IBM Corp, Armonk, NY, United States).

### 2.7 Ethical aspects of the study

The study was conducted in accordance with the Declaration of Helsinki, and approved by the Bioethics Committee of the University of Salamanca (ID507). The Bioethics Committee of the University of Salamanca has favorably agreed to carry out this research with registration number ID507, complying with the ethical requirements for its execution on 3 February 2021.

## 3 Results

### 3.1 Descriptive statistics

The final sample of the study comprised *n* = 107 individuals.

Tables 1–3 provide a comprehensive overview of the descriptive data related to the sociodemographic variables and the variables under consideration in the study. The data show that the median age of patients is 55 years, while the median age of primary caregivers is 57 years. There is an overwhelming majority of women, accounting for 99.1% of the sample’s gender distribution. Regarding the educational level of the study subjects, primary education predominates, with 43% of the sample. In terms of occupation, the majority are employed by others (43.9%). Additionally, almost half of the sample is married (48.6%). Lastly, concerning the stage of the oncological disease, we observed a higher incidence of stages I and II, with 31.8 and 35.5%, respectively.

**Table 1:** Descriptive analysis of study variables I.

Variable	Median (M)	Interquartile range (RIQ)
Age	55	15
Caregiver age	57	16
Months since diagnosis	12	22
Barthel index	100	20
Lawton Brody scale	8	2
ECOG	2	2
ZARIT questionnaire	15	37

**Table 2:** Descriptive analysis of study variables II.

Variable		Frequency	Percentage
Gender	Man	1	0.9%
	Woman	106	99.1%
Marital status	Single	22	20.6%
	Married	52	48.6%
	Separated	19	17.7%
	Widowed	14	13.1%
Level of education	Primary education	46	43%
	Secondary education	33	30.8%
	Higher education	28	26.2%
Profession	Self-employed	9	8.4%
	Employed by others	47	43.9%
	Not in employment	25	23.4%
	Other	26	24.3%
Stage of cancer disease	Stage I	34	31.8%
	Stage II	38	35.5%
	Stage III	18	16.8%
	Stage IV	17	15.9%

**Table 3:** Average descriptive analysis of the financial costs of cancer disease.

Extraordinary expenditure	*N*	Minimum	Maximum	Media	SD
Extraordinary expenditure on pharmaceuticals for oncological disease	107	0	2,100	316.82	284.998
Extraordinary expenditure on orthopedic equipment for oncological disease	107	0	3,300	487.85	636.427
Extraordinary expenditure on home help	107	0	3,300	140.19	533.033
Extraordinary expenditure on housing adaptation or transfer to a hospital	107	0	3,300	566.36	769.090
Amount of annual net household income prior to diagnosis	107	3,000	150,000	19962.62	15452.255
Amount of annual net household income during the last fiscal year	107	2,100	33,000	16785.98	8974.339

In the descriptive analysis of the study variables, it is notable that patients exhibit a median Barthel Index score of 100 points (indicating independence in daily activities), 8 points on the Lawton and Brody scale (indicating independence in instrumental activities of daily living), and 2 points on the ECOG scale. Furthermore, analyzing the primary caregiver’s level of burden, a median ZARIT questionnaire score of 15 points was obtained, indicating a high level of burden.

The descriptive analysis of the economic expenses related to the oncological disease is presented below. These results are depicted in Table 3. The average total expenditure amounts to 1511.22 euros per year, expenses solely attributed to the diagnosis of cancer. It is also observed that the average annual income before diagnosis is 19962.62 euros, while after the onset of the oncological disease, it decreases by 15.91%, resulting in an average annual income reduction of 16785.98 euros.

Upon further analysis of different components, a distinction has been made in expenses related to the acquisition of orthopedic material, home assistance, pharmacy, the need for hiring a third party, and the necessity for home adaptation or hospital transfer. The data are presented in Table 4.

**Table 4:** Descriptive analysis of cancer-related economic expenditure.

Expenditure situations		Frequency	Percentage
Expenditure on orthopedic equipment related to oncological disease	No expenditure	38	35.5%
	Less than 600 euros	36	33.6%
	From 600 to 1,200 euro	25	23.4%
	From 1,201 to 1,800 euro	4	3.7%
	From 1,801 to 2,400 euro	2	1.9%
	From 2,401 to 3,000 euro	1	0.9%
	More than 3,000 euro	1	0.9%
Expenditure on home help related to oncological disease	No expenditure	96	89.7%
	Less than 600 euros	7	6.5%
	From 600 to 1,200 euro	0	–
	From 1,201 to 1,800 euro	2	1.9%
	From 1,801 to 2,400 euro	0	–
	From 2,401 to 3,000 euro	0	–
	More than 3,000 euro	2	1.9%
Expenditure on home adaptation or hospital transfer related to the oncological disease	No expenditure	29	27.1%
	Less than 600 euros	47	43.9%
	From 600 to 1,200 euro	17	15.9%
	From 1,201 to 1800 euro	6	5.6%
	From 1.801 to 2,400 euro	2	1.9%
	From 2,401 to 3,000 euro	1	0.9%
	More than 3,000 euro	5	4.7%
Expenditure on necessary third parties related to the oncological disease	No expenditure	77	72%
	Less than 600 euros	11	10.3%
	From 600 to 1,200 euro	9	8.4%
	From 1,201 to 1,800 euro	5	4.7%
	From 1,801 to 2,400 euro	1	0.9%
	From 2,401 to 3,000 euro	1	0.9%
	More than 3,000 euro	3	2.8%
Pharmacy expenditure related to oncological disease	No expenditure	20	18.7%
	Less than 600 euros	74	69.2%
	From 600 to 1,200 euro	12	11.2%
	From 1,201 to 1,800 euro	0	–
	From 1,801 to 2,400 euro	1	0.9%
	From 2,401 to 3,000 euro	0	–
	More than 3,000 euro	0	–

### 3.2 Analytical statistics

Subsequently, analytical statistics were conducted, initially involving a correlation analysis of the variables under scrutiny.

The first correlation analysis is presented in Table 5, wherein:

**Table 5:** Correlation analysis.

Rho de Spearman		Age	Barthel	Lawton Brody	ECOG	ZARIT
Age	Correl. coefficient	1,000	−0.534^**^	−0.571^**^	0.505^**^	0.341^**^
	Sig.	–	<0.001	<0.001	<0.001	0.003
Barthel	Correl. coefficient	−0.534^**^	1,000	0.881^**^	−0.799^**^	−0.557^**^
	Sig.	<0.001	–	<0.001	<0.001	<0.001
Lawton y Brody	Correl. coefficient	−0.571^**^	0.881^**^	1,000	−0.815^**^	−0.530^**^
	Sig.	<0.001	<0.001	–	<0.001	<0.001
ECOG	Correl. coefficient	0.505^**^	−0.799^**^	−0.815^**^	1,000	0.520^**^
	Sig.	<0.001	<0.001	<0.001	–	<0.001
ZARIT	Correl. coefficient	0.341^**^	−0.557^**^	−0.530^**^	0.520^**^	1,000
	Sig.	0.003	<0.001	<0.001	<0.001	–

^**^The correlation is significant at the 0.01 level (two-way).

A direct correlation is established between Barthel, Lawton Brody, ECOG, and Zarit scores (*p* < 0.005). This implies that higher levels of dependency are associated with a poorer quality of life among cancer patients and a heightened level of burden for the primary caregiver.

A direct correlation is also identified between age and levels of dependency and caregiver burden among cancer patients (*p* < 0.005). In essence, the older the patient, the higher the levels of dependency observed, consequently leading to increased caregiver burden.

Finally, a secondary regression analysis was performed, aiming to ascertain the relationship between expenditure levels and the various variables under examination. The following results were obtained:

A direct relationship exists between the level of dependency, as measured by the Barthel index, and expenses related to home assistance (*r* = −0.488; *p* < 0.05), as well as with expenses associated with home adaptation (*r* = −0.252; *p* < 0.05).

Similarly, a direct correlation is observed between the level of dependency in instrumental activities of daily living, measured via the Lawton Brody scale, and expenses related to home assistance (*r* = −0.476; *p* < 0.05).

Further exploration of the relationship between variables was conducted through a linear regression study, with total extraordinary expenditure and expenditure in each of the studied areas (pharmacy, orthopedics, home assistance, and housing) serving as dependent variables. This analysis yielded statistically significant relationships:

- Total expenditure and level of dependency (Barthel Index), *p* = 0.032.
- Total expenditure and patient functional status (ECOG), *p* = 0.045.
- Expenditure on orthopedic material and patient functional status (ECOG), *p* = 0.025.
- Expenditure on home care and level of dependency (Barthel index), *p* = 0.043.
- Expenditure on housing and level of dependency (Barthel index), *p* = 0.033.

## 4 Discussion

The main objective of this study was to examine the socioeconomic consequences of breast cancer on patients and their families. Cancer represents one of the most significant health issues worldwide (16–18), affecting individuals to varying degrees and incurring additional expenses that impact their daily lives. This study provides evidence of the costs associated with cancer, with the most significant ones being those related to the acquisition of pharmaceutical materials (€316.82 per year), orthopedic materials (€487.85 per year), home assistance (€140.19 per year), and hospital transportation (€566.36 per year), along with a decrease in income by 15.91%. It is worth noting that previous literature has analyzed these same categories in studies of similar characteristics in other countries (19–21). A clear pattern emerges from the variables analyzed in various studies, which is similar to our findings. These variables include gender, sex, cancer type, stage, educational level, place of residence, employment status, annual household income, months elapsed since diagnosis, disability and type (22–24).

It is crucial to highlight the importance of financial support for cancer patients. Numerous studies, including this one, have demonstrated that the public health system fails to adequately meet the financial needs of patients (25, 26). This shortcoming is particularly evident in Spain, where Dependency Law 39/2006, designed to aid individuals with intensive support needs, has an excessively long resolution period, sometimes extending up to 6 months. This bureaucratic delay not only hinders access to necessary resources at critical times but also exacerbates the financial strain on families already dealing with additional costs associated with the disease, such as uncovered medical expenses, transportation, and home adaptations.

The most tragic aspect of this situation is that many patients who urgently require this support pass away before receiving the necessary assistance. This unfortunate outcome highlights a systemic failure in delivering essential services, underscoring the need for immediate reforms in the process of granting aid under the Dependency Law. The delay in resolving these applications not only has a devastating impact on the quality of life of patients and their families but also perpetuates socioeconomic inequality by leaving the most vulnerable individuals unattended. A review and streamlining of these procedures are imperative to ensure that the public health system fulfills its goal of providing effective and timely support to those who need it most.

The quantity of income for households is greater when the patient was employed or self-employed prior to their diagnosis, as opposed to receiving any disability, retirement, or other benefits. There are many patients in our sample who, due to their diagnosis, are not able to continue working in their own company despite being self-employed and are required to continue paying contributions and other expenses.

Certainly, a cancer diagnosis is accompanied by a decrease in functionality, including impairments in both basic and instrumental activities of daily living (ADLs), and an associated increase in dependence. These issues have been observed in studies that examine a range of aspects, such as patient mobility and levels of autonomy (25, 26). This finding is of significant importance for our research since our results indicate that higher scores on scales assessing patient autonomy correspond to greater healthcare expenditures.

Difficulties arise when deciding to become the primary caregiver for a patient due to the risk of developing claudication, Burnout Syndrome, or overload (16, 27). This is particularly relevant as our study has shown that caregiver overload has a direct impact on the family’s expenditure.

Having said all of this, one of the most crucial findings was the identification of a link between cancer and household income. The lower an individual’s socio-economic status, the more significant the detrimental impact on their prognosis (28). We can therefore infer that the key factor contributing to the reduction in income is the cancer itself and its progression.

To assess the socio-economic effects on other pathologies, we conducted a comprehensive literature review and identified a significant study in our country that addresses a variety of neurodegenerative conditions.

A study by Garcés et al. (29) featured an extensive patient cohort with various neurodegenerative diseases, such as Alzheimer’s and other dementias, Parkinson’s disease, multiple sclerosis (MS), neuromuscular disorders, and amyotrophic lateral sclerosis (ALS).

The findings closely resemble those of our study. Both neurodegenerative and oncological diseases inflict a substantial socio-economic burden on patients and their loved ones. This burden is shaped by numerous factors, including pharmacy costs, assistive products, home modifications, transportation, and more.

It is worth noting that all illnesses entail significant expenses for their sufferers and families. Regrettably, these increased costs are not covered by the national healthcare system.

The study conducted by Mutyambizi et al. (30) on diabetes at two public hospitals in South Africa affirms that patients are accountable for up to 50% of healthcare expenses, leading to disparities between poor and affluent families and acting as a catastrophic determinant of patients’ health.

This study underscores how various pathologies generate costs that are indirectly borne by patients and their families.

The study undertaken by Russella and Gilson (31) examined the direct relationship between health and economic impact in diverse households. The results indicated that chronic or serious illnesses result in high costs for families and may negatively affect their means of subsistence.

He concluded that in Sri Lanka, where there is a free public health service, household expenditures caused by severe illnesses were indirect costs that arose from the illness and were not paid for by the public service.

The study by Chuma et al. (32) is significant for demonstrating the direct and indirect costs households can incur due to chronic illness. This can exacerbate the socio-economic situation of the patient and family, resulting in decreased well-being.

Financially-stricken households with chronic illnesses are more common among lower-income families, who primarily use the sale of family assets and real estate to fund healthcare costs (32).

Regrettably, the significant impact on quality of life is often overlooked.

Public policies must address situations where not only the disease is significant from a health and biological standpoint, but also from social, socio-economic, and financial perspectives.

Furthermore, it is important to emphasize a noteworthy finding from our study, revealing a direct correlation between individuals’ level of dependence and increased financial expenses. This contrasts with the results of Garcia et al. (33) previous studies. Where inconsistent data was observed, a statistically significant relationship could not be established. The studies differed only in their anatomopathological diagnosis; our study solely involved patients diagnosed with breast cancer, whereas the analyzed study included all types of cancer without stratification by diagnosis. This finding may serve as a foundation for future studies.

### 4.1 Limitations

#### 4.1.1 Direct and indirect causality

We were only able to observe this in patients already diagnosed with oncological disease, but not from the beginning, rendering our study incomplete as we cannot ascertain with total accuracy the evolution of the impact caused.

#### 4.1.2 Lack of evidence and reliability

The bibliographic search conducted has been scarcer than expected, due to resource constraints and the scarcity of scientific evidence in some cases.

#### 4.1.3 Ethics and morality

Money as a taboo subject. By this point, I mean that a significant limitation has been the reluctance of many patients, especially the older adult or women in more traditional settings, to discuss their income. Consequently, many questionnaires had to be discarded, leading to a decrease in the obtained sample size.

Despite findings from multiple studies supporting our hypothesis that cancer deteriorates the functionality of the sufferer and generates additional expenses, we recognize that oncological disease is influenced by multiple factors, with cancer itself being the primary driver of the socioeconomic impact.

The widespread lack of awareness is the main obstacle to addressing this significant problem that affects us all, directly or indirectly, diminishing quality of life.

Based on the findings, it’s imperative to further investigate the economic shortcomings stemming from diagnoses of serious illnesses like cancer. This exploration is crucial for gaining a precise understanding of the origins of these financial limitations, with a particular focus on distinguishing between different types of oncological diagnoses. Once these deficits and their causes are identified, it becomes essential to implement necessary changes aimed at alleviating the severe impact of diseases like cancer on individuals’ lives. This includes ensuring that economic challenges resulting from the illness do not exacerbate the already significant burden faced by patients and their families.

## 5 Conclusion

The study has identified various socio-economic challenges encountered by oncology patients, including expenses related to pharmacy, parapharmacy, orthopedic materials, accompanying services, external professional caregivers, or transportation to the hospital. Additionally, the degree of dependency or autonomy affected by the oncological disease impacts socio-economic status, as incapacity often leads to the abandonment of occupations, resulting in a decrease in income. Furthermore, household income experiences a significant reduction when primary caregivers experience overload.

In summary, the overarching conclusion of the study is that the additional expenses incurred by breast cancer patients are primarily attributable to the diagnosis of breast cancer itself.

## Data availability statement

The original contributions presented in the study are included in the article/Supplementary material, further inquiries can be directed to the corresponding authors.

## Ethics statement

The studies involving humans were approved by the Bioethics Committee of the University of Salamanca. The studies were conducted in accordance with the local legislation and institutional requirements. The participants provided their written informed consent to participate in this study.

## Author contributions

EF-R: Conceptualization, Investigation, Methodology, Writing – original draft. RT-T: Formal analysis, Resources, Software, Writing – original draft. AG-M: Conceptualization, Data curation, Methodology, Writing – review & editing. CS-G: Conceptualization, Data curation, Validation, Writing – review & editing. SS-G: Data curation, Investigation, Writing – review & editing. MR-G: Formal analysis, Validation, Writing – review & editing. EF-S: Formal analysis, Methodology, Writing – review & editing.

## Supplementary material

Supplementary material 1 Bioethics committee approval.

Supplementary material 2 Informed consent and information sheet for participants.

Supplementary material 3 STROBE checklist.

Supplementary material 4 Statistical data study.

Supplementary material 5 Questionnaire on the socio-economic impact of breast cancer disease on patients and their families.
